# Modeling Radiation-Induced Epithelial Cell Injury in Murine Three-Dimensional Esophageal Organoids

**DOI:** 10.3390/biom14050519

**Published:** 2024-04-25

**Authors:** Latisha Carswell, Deepa M. Sridharan, Lung-Chang Chien, Wataru Hirose, Véronique Giroux, Hiroshi Nakagawa, Janice M. Pluth

**Affiliations:** 1 Mercer University School of Medicine, Macon, GA 31207, USA; latisha.tamika.pryor.carswell@live.mercer.edu; 2 Soley Theraperutics, Inc., South San Francisco, CA 94080, USA; dsridharan@berkeley.edu; 3 Department of Epidemiology and Biostatistics, University of Nevada, Las Vegas, NV 89154, USA; lung-chang.chien@unlv.edu; 4 Herbert Irving Comprehensive Cancer Center, Columbia University, New York, NY 10032, USA; wh2564@cumc.columbia.edu (W.H.); hn2360@cumc.columbia.edu (H.N.); 5 Department of Immunology and Cell Biology, Universite de Sherbrooke, Sherbrooke, QC J1E 4K8, Canada; veronique.giroux@usherbrooke.ca; 6 Digestive and Liver Diseases Research Center, Organoid & Cell Culture Core, Columbia University, New York, NY 10032, USA; 7 Health Physics and Diagnostic Sciences, University of Nevada Las Vegas, Las Vegas, NV 89154, USA

**Keywords:** esophageal, 3D organoid culture, DNA damage, high-LET radiation

## Abstract

Esophageal squamous cell carcinoma (ESCC) is a deadly consequence of radiation exposure to the esophagus. ESCC arises from esophageal epithelial cells that undergo malignant transformation and features a perturbed squamous cell differentiation program. Understanding the dose- and radiation quality-dependence of the esophageal epithelium response to radiation may provide insights into the ability of radiation to promote ESCC. We have explored factors that may play a role in esophageal epithelial radiosensitivity and their potential relationship to ESCC risk. We have utilized a murine three-dimensional (3D) organoid model that recapitulates the morphology and functions of the stratified squamous epithelium of the esophagus to study persistent dose- and radiation quality-dependent changes. Interestingly, although high-linear energy transfer (LET) Fe ion exposure induced a more intense and persistent alteration of squamous differentiation and 53BP1 DNA damage foci levels as compared to Cs, the MAPK/SAPK stress pathway signaling showed similar altered levels for most phospho-proteins with both radiation qualities. In addition, the lower dose of high-LET exposure also revealed nearly the same degree of morphological changes, even though only ~36% of the cells were predicted to be hit at the lower 0.1 Gy dose, suggesting that a bystander effect may be induced. Although p38 and ERK/MAPK revealed the highest levels following high-LET exposure, the findings reveal that even a low dose (0.1 Gy) of both radiation qualities can elicit a persistent stress signaling response that may critically impact the differentiation gradient of the esophageal epithelium, providing novel insights into the pathogenesis of radiation-induced esophageal injury and early stage esophageal carcinogenesis.

## 1. Introduction

Radiation exposure remains a pervasive concern in modern society, with implications ranging from medical diagnostics and therapeutics to occupational hazards and environmental risks. Of particular interest is the intricate relationship between dose and radiation quality and its impact on cancer development, a connection whose underlying mechanisms are still being deciphered. More recent studies have observed that the cellular and molecular changes differ following exposure to low vs. high doses of radiation, with doses below 0.2 Gy showing biological changes in the opposite direction to those observed with doses higher than 0.5 Gy, with the hypothetical transition dose being ~0.4 Gy (summarized in [1]). Thus, extrapolating from high-dose data to understand low-dose radiation effects may lead to incorrect conclusions. It has also been observed that cellular and gene expression responses differ depending upon radiation quality, which is perhaps explained in part by the fact that low-LET radiation produces more indirect effects, whereas high-LET radiation produces more direct effects [2,3]. 

In this paper, we chose to investigate the persistent cellular effects of both low and high doses of radiation, as well as radiation quality, using a mouse 3D esophageal tissue model system. Esophageal tissue is of special interest, due to its radiation-sensitivity and mouse cells and tissue models have been widely used to investigate esophageal cancer risk due to their molecular similarities to human cells [4]. In particular, similarities between mouse and human cells have been observed for several radiation-induced gene responses [5]. However, discordances between mouse and human cells have been noted, which limits the ability to fully extrapolate animal findings to humans. Even given this caveat, important findings can be made utilizing mouse models systems which will help advance our understanding of the underlying nature of esophageal cells’ inherent radiosensitivity. 

Our investigation focused on elucidating the mechanisms underlying radiation-induced tissue injury in 3D mouse esophageal tissues, comparing the effects of high- and low-dose radiation. Additionally, we explored the dose-response relationship using both low- and high-linear energy transfer (LET) radiation to understand how different radiation qualities influence carcinogenic processes. Through this comprehensive approach, we aimed to provide insights into radiation protection strategies and enhance the efficacy of cancer therapies. Moreover, the findings from this study can contribute to refining treatment approaches for radiation-induced esophageal cancers, potentially improving patient outcomes and quality of life. By incorporating both a low (0.1 Gy) and tenfold higher (1 Gy) dose, as well as low- and high-LET radiation, our multidimensional approach unravels the complex interplay between dose and radiation quality effects on cancer development, thus advancing our understanding of esophageal radiation sensitivity.

**Figure 1:**
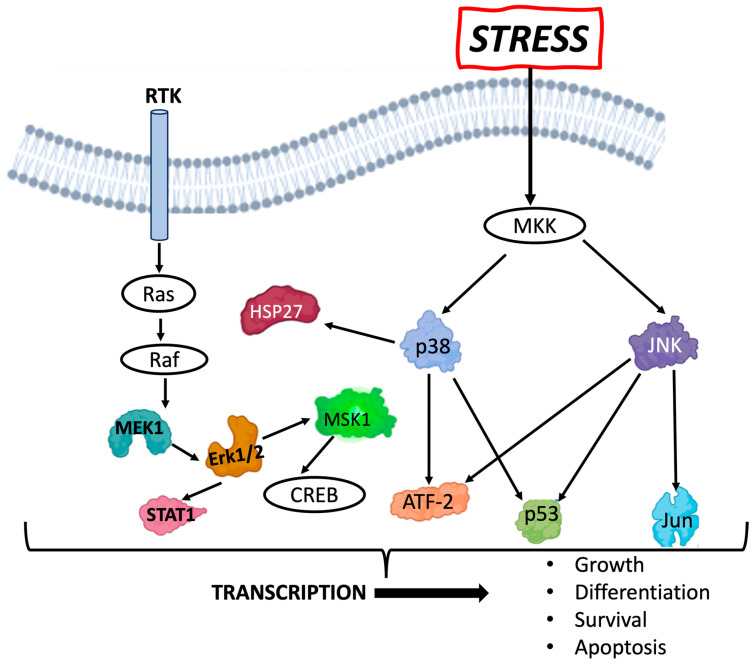
Various phospho-proteins activated through the receptor tyrosine kinases (RTKs) or via stress leading to transcription and to multiple possible cellular fates. Colored proteins are phospho-proteins studied in this work; the relationships between these proteins are shown by arrows. Created with BioRender.com.

The MAPK/ERK pathway (Figure 1), also referred to as the Ras/Ref/MEK/ERK pathway, communicates signals from the receptor tyrosine kinase receptor on the surface of the cell to the nucleus. The ERK pathway can be activated by mitogenic stimuli, such as growth factors and cytokines, and plays a role in regulating cell growth, survival, and differentiation. The various phospho-proteins in this pathway are activated under assorted stressors, such as UV or ionizing radiation exposure, and play a role in the transcription of genes defining cell fate. For example, the MEK/ERK pathway has been shown to contribute to an anti-apoptotic pathway following radiation, providing cells with radioresistance [6,7]. ERK activation has been shown to trigger cell proliferation and to provide a survival advantage [8,9], although it has also been known to promote cell death under other conditions, depending upon its location and the extent of activation [10]. In nasopharyngeal cancer cells, inhibition of HSP27 expression decreased cell survival and viability and increased apoptosis, highlighting its potential function in radioresistance [11]. Activation of p53 at Ser15 has been implicated in contributing to miR-34a transcription in mammary epithelial cells and thought to play a role in oncogene senescence. MSK1 is activated by MAPK2/ERK2 and leads to CREB phosphorylation and the transcription of genes related to early radiation response [12,13]. JNK and p38 MAPK are poorly activated by growth factors but respond strongly to stress signals and have also been associated with apoptotic cell death. Thus, activation of the proteins in the MAPK/ERK pathway may have various context-dependent effects on cell fate determination.

Universal changes observed in cancer include alterations in tissue structure and morphology. Abnormal uncontrolled cell proliferation and differentiation are key characteristics of cancer [14]. DNA damage signaling is also increased and shows persistent higher levels [15]. Stress signaling has been linked to higher levels of radiation-induced reactive oxygen species (ROS) and may in part explain the persistent higher DNA damage levels [16,17]. Quantitatively determining how dose and radiation quality may influence tissue changes and DNA damage response and stress phospho-protein signaling may aid in defining the underlying mechanisms of the alterations leading to esophageal cancer. To better understand esophageal tissue radiosensitivity, prior studies have exposed immortalized (non-transformed) normal human esophageal cell lines in monolayer cultures to various radiation qualities and doses. Most of these studies, assayed at early time points (≤8 h), or up to three days post-exposure in one study, have revealed that high-linear energy transfer (LET) particles have a greater biological effect, showing enhanced EMT, Smad7 foci, and cell migration [18,19,20]. However, the longer-term (>1 week) post-radiation impacts upon esophageal epithelial structure and stress-induced signaling molecules have not been explored to date. Herein, we utilized for the first time the murine 3D esophageal epithelial organoid model that recapitulates squamous esophageal epithelia under homeostasis and pathologies, both benign and malignant [21], to evaluate how exposure to cesium and high-LET Fe at a low vs a higher dose (0.1 and 1 Gy) may impact long-term (9 days post-radiation) normal esophageal epithelial integrity. In 3D mouse esophageal organoids, we quantified changes in epithelial architecture, DNA damage levels, and the mitogen-/stress-activated protein kinase (MAPK/SAPK)-mediated signaling pathway following radiation exposure to provide insights into radiation changes that may increase esophageal cancer risk.

## 2. Materials and Methods

### 2.1. Esophageal Three-Dimensional (3D) Organoids

C57/BL6 mice (8–12-week-old, male and female) (Jackson laboratory, Bar Harbor, ME, USA) received humane care and underwent procedures according to a protocol approved by the Institutional Animal Care and Use Committee (IACUC) at Columbia University. Mice were sacrificed and esophageal epithelial cells harvested to generate esophageal 3D organoids as described in [21]. In brief, 5000 cells were seeded in 50 µL Matrigel^TM^ (BD Biosciences, San Jose, CA, USA) per well in 24-well plates and fed with 500 µL of DMEM/F12 supplemented with 1× Glutamax, 1× HEPES, 1× N2, 1× B27, 0.1 mM N-acetyl-L-cysteine (Sigma-Aldrich, Burlington, MS, USA), 50 ng/mL mouse recombinant epidermal growth factor (R&D Systems, Minneapolis, MN, USA), 10 µM Y276 (Tocris Biosciences, Bristol, UK), and 2.0% Noggin/R-Spondin-conditioned medium. Organoids were recovered by digesting Matrigel with Dispase I (BD Biosciences, San Jose, CA, USA; 1 U/mL), fixed overnight in 4.0% paraformaldehyde, and embedded in 2.0% Bacto-Agar: 2.5% gelatin prior to paraffin embedding. The resulting paraffin-embedded sections were subjected to hematoxylin and eosin (H&E) and immunofluorescence (IF) staining.

### 2.2. Radiation Exposure 

Radiation exposures (both Cs and Fe 600 MeV/u) were performed at NSRL at Brookhaven National Laboratory, Long Island, NY, USA. The Fe 600 MeV/u 0.1 Gy exposure was performed at a dose rate of 8.2 cGy/min, and the 1 Gy exposure was performed at a dose rate of 95.7 cGy/min. Cs exposures utilized a 10× attenuator that provided a dose rate of 10.65 cGy/min for the 0.1 Gy dose and a 2X attenuator that provided a dose rate of 61.13 cGy/min for the 1 Gy dose. Organoids were irradiated on day 2 post-seeding and grown for nine additional days following irradiation. 

### 2.3. Luminex Assay for Stress Pathway Phospho-Protein Expression

Stress pathway phospho-protein expression was quantified using a Millipore Luminex-based kit (cat. no. 48-660MAG) using a 96-well plate format and cell lysates from organoid cultures. The phosphorylated proteins detected included phosphorylated ERK/MAP kinase 1/2 (Thr 185/Tyr187), JNK (Thr183/Tyr185), MEK1 (Ser217/221), MSK1 (Ser212), ATF2 (Thr71), p53 (Ser15), HSP27 (Ser78), c-Jun (Ser73), and p38 (Thr180/Tyr182). Approximately 1 mL of lysis buffer was added per 10^7^ cells and stored at −80 °C until assayed. The protein levels from each sample were quantified using a nanodrop instrument, and equal amounts were added to define phospho-protein expression. The samples were run on a Bio-Plex 200 (BioRad, Hercules, CA) system supplied by the UNLV Cellular and Molecular Brain Research Laboratory. Samples were run in duplicate.

### 2.4. Immunofluorescence Staining for 53BP1 Foci and Involucrin

Organoid cultures were fixed with 10% formalin, embedded and sectioned to slides, then warmed to 60 °C and treated with a xylene wash and then a series of EtOH dehydration series washes (100, 95, 80, and 70%). Slides were rinsed in water then placed in a pressure cooker in Citric acid solution (pH 6.0) for 2 h. Slides were washed in water and then in PBS and treated with a 5% BSA block for 1 h. Rabbit 53BP1 antibody (Behthyl labs, Montgomery, TX, IHC-00001) was diluted 1:1000 in 1% BSA and left overnight at 4C. The following day, slides were washed in PBS and the secondary antibody (Goat anti-rabbit Alexa 488, A28175, 1:400, Invitrogen, Waltham, MA, USA) was applied at a 1:600 dilution in 1% BSA and incubated for 2 h at room temp. Slides were washed in PBS and water and were then 4′,6-diamidino-2-phenylindole (DAPI) counterstain added and slides mounted with vectashield and coverslipped. Slides were scored blindly, identifying cells within organoids that were positive for 53BP1 staining, and the percentage of foci-positive cells per organoid for each treatment quantified. Likewise, slides were incubated with anti-Involucrin (IVL) monoclonal antibody (I9018, 1:100; Sigma-Aldrich, Burlington, MA, USA) overnight at 4 °C. Alexa Fluor^TM^ Plus 488-conjugated affinity-purified anti-mouse IgG (A766, 1:400, Invitrogen, Waltham, MA, USA) was used for IVL signal detection by incubation at 37 °C for 2 h, and cell nuclei were counterstained by DA-containing mounting medium (H-1500-10; Vector Laboratories, Newark, CA, USA). Stained objects were examined with a KEYENCE automated high-resolution microscope BZ-X800 and imaged with a digital camera. Relative signal intensity was analyzed using ImageJ software verison 2.14.0 [22]. For IVL, relative intensity was measured by multiplying the average intensity by the area stained and dividing by the number of cells. 

### 2.5. Statistical Analysis

To compare measurements of thickness and foci for the 0 Gy, 0.1 Gy, and 1 Gy doses, we constructed two general linear models. Both models incorporated radiation and dose as the main predictors along with their interaction. In particular, the model for thickness included a slide effect as a covariate. Utilizing the fitted models, we conducted multiple comparisons with Bonferroni adjustments to mitigate compound uncertainty. The mean difference in each comparison was determined by the least squares mean difference. The adjusted CIs of mean differences were also computed. Data analyses were performed using SAS version 9.4 (SAS Institute, Cary, NC, USA). The significance level was set to 0.05. Comparisons of IVL intensity were analyzed using one-way ANOVA.

## 3. Results 

### 3.1. Radiation Quality and Dose Effects in Mouse Esophageal Organoids

**Figure 2:**
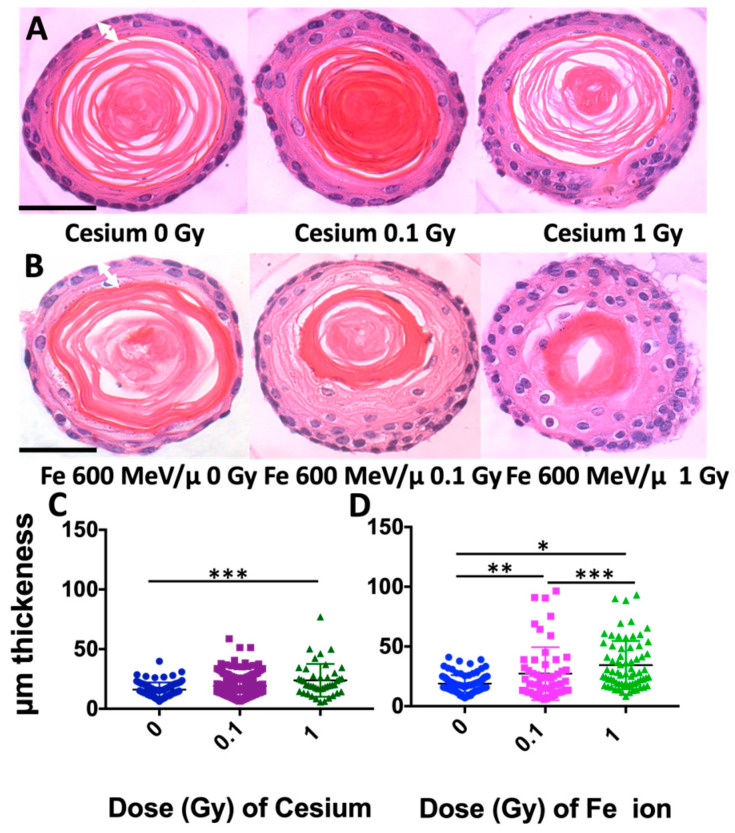
Radiation quality- and dose-dependent changes in the esophageal epithelial differentiation gradient in mouse 3D organoid models. H&E-stained mouse organoids were assessed for the thickness of the outer non-cornifying cell layers (examples shown with white arrows in 0 Gy samples) following exposure to indicated doses (0–1 Gy) of Cs (**A**) or Fe 600 MeV/µ (**B**). Note that there was a significant increase in thickness of the non-cornifying epithelial cell layer at 1 Gy as compared to 0 Gy (control) in (**C**,**D**). High-LET radiation Fe 600 MeV/µ induced a significant dose-dependent increase in thickness of the non-cornifying epithelial cell layer in (**D**). Scale bar, 50 µm in (**A**). * *p*-value < 0.0001, ** *p*-value < 0.001, *** *p*-value < 0.02.

To define how radiation quality and dose influence normal organoid formation, mouse 3D esophageal organoids were grown in Matrigel, irradiated and changes in morphology quantified. On day two post-initiation, organoids were irradiated with a 0.1 or 1 Gy dose of either Cs or high-LET Fe 600 MeV/µ. Cells were incubated for nine additional days, allowing the formation of esophageal organoids. On day eleven post-initiation, organoids were collected, formalin-fixed, paraffin-embedded, and sectioned for hematoxylin and eosin (H&E) staining to detect possible epithelial structural changes based on dose or radiation quality. Although radiation did not induce cellular atypism within the organoids, there was an expansion of basaloid cells as well as the intermediate differentiating (spinous) cell layer with radiation exposure, suggesting a perturbation in the squamous cell differentiation gradient (Figure 2A,B). Such a morphological epithelial change in the radiation-exposed organoids was assessed by measuring the thickness of the non-cornifying outer layer, constituting both the basal and suprabasal layers (Figure 2A,B, regions measured noted in 0 Gy samples with white arrows). Both Cs and Fe ions showed an increase in thickness (µm) of the outer cellular layers with increasing doses; however, for Cs, a significant change was only observed between the 0 Gy and 1 Gy samples (Figure 2C, significant by 8.10 μm, 95% confidence interval [CI] = 0.70, 15.51; *p*-value = 0.0199). Furthermore, after exposure to high-LET (Fe 600 MeV/µ), a significant increase in the outer layer thickness at both 0.1 and 1 Gy doses as compared to the untreated control (0 Gy) was observed (Figure 2D). Organoids were also scored for differences in diameter, but no significant differences were noted for this endpoint. 

### 3.2. Dose–Response MAPK/SAPK Stress Signaling in Mouse Esophageal Organoids in Response to Radiation Exposure

**Figure 3:**
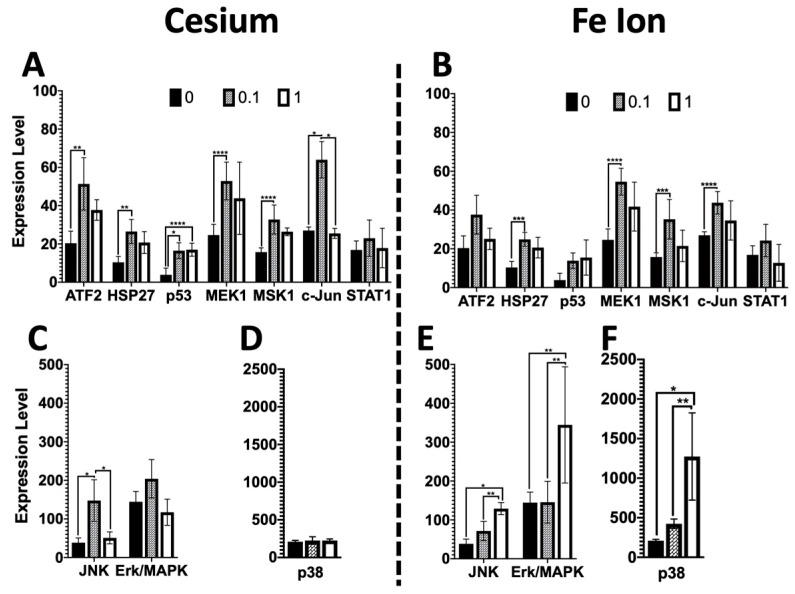
Dose and radiation quality comparison of phospho-protein expression changes in the stress pathway in mouse esophageal organoids. Many phospho-proteins for both radiation qualities showed a trend of increased expression with the lower 0.1 Gy dose radiation as compared to the control or a higher 1 Gy dose (**A**–**C**). For Cs exposed cells no change was observed with dose for p38 (**D**). A few phospho-proteins showed a significant dose response, including JNK and p38 for Fe ions (**E**,**F**) and p53 for Cs (**A**). Significant *p*-values are noted: * *p*-value < 0.0001, ** *p*-value < 0.002, *** *p*-value < 0.01, **** *p*-value < 0.05.

A multiplexed assay was used to define the expression levels of 10 phospho-proteins in the MAPK/SAPK stress pathway (Figure 1) nine days following radiation exposure. The phospho-sites assayed within the stress pathway included ERK/MAP kinase 1/2 (Thr185/Tyr187), STAT1 (Tyr701), JNK (Thr183/Tyr185), MEK1 (Ser217/221), MSK1 (Ser212), ATF2(Thr71), p53 (Ser15), HSP27 (Ser78), c-Jun (Ser73), and p38 (Thr180/Tyr182). The fold change in levels of phospho-proteins in exposed organoids as compared to control was determined for both Cs and Fe600 MeV/µ. For both Cs and Fe ions, the induction of most phospho-proteins (ATF2, HSP27, MEK1, MSK1, and c-Jun) appeared to be greatest at the lower 0.1 Gy dose as compared to the 1 Gy dose and non-exposure control (Figure 3). This pattern was also observed for Cs with JNK and ERK/MAPK phospho-proteins (Figure 3, cesium, lower graph). However, for high-LET Fe ion-exposed samples, the JNK and ERK/MAPK phospho-protein levels were higher following the 1 Gy dose (Figure 3, Fe ions, lower graph), revealing a dose-dependent response. P38 was unique in showing no real change from the control for Cs (Figure 3C) and a very large dose-dependent increase with Fe ion exposure (Figure 3D). A dose-dependent increase was also observed for both radiation qualities with p53 (Figure 3A,B), although it was statistically significant only for Cs.

### 3.3. Persistent DNA Damage Foci Reveal a Radiation Quality- and Dose-Dependent Pattern

**Figure 4:**
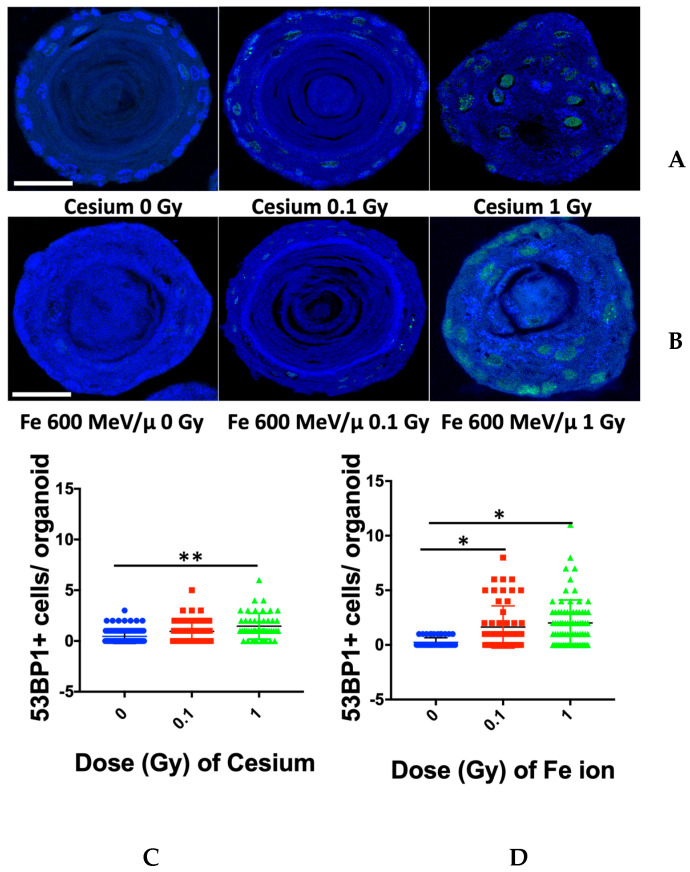
Nine days post-radiation, organoids showed residual signs of damage, as noted by persistent 53BP1 foci, (**A**,**B**), representative images. Representative images of mouse esophageal organoids for each treatment are shown (top 2 rows). 53BP1 is labeled with Alexa 488 (green), and nuclei are counterstained with DAPI (blue). The number of foci-positive cells per organoid were scored for each dose and radiation quality and graphed. Cs (**C**) or Fe ions (**D**). * *p*-value < 0.0001, ** *p*-value < 0.002. Scale bar, 50 µm.

To define whether persistent DNA damage signaling could be observed in 3D organoid structures nine days post-radiation exposure, a DNA damage marker, 53BP1, was used to stain sectioned organoids to quantify the percentage of DNA damage foci-positive cells per organoid (Figure 4A,B, representative images). 53BP1 is a known DNA repair factor that localizes to DNA DSBs and promotes NHEJ [23] repair. Nine days post-exposure to Cs (Figure 4C) or Fe ions (Figure 4D), the number of 53BP1-positive cells for each organoid was quantified. We observed a dose-dependent increase for both radiation qualities; however, for cesium, the only significant difference in the number of 53BP1 positive organoids was for the 1 Gy dose as compared to the control, with an increment of 1.00 (95% CI = 0.24, 1.76; *p*-value = 0.0018). Significant differences were noted in the number of 53BP1-positive cells per organoid between the control and the 0.1 and 1 Gy dose of high-LET Fe ion-exposed organoids (by 1.41 foci-positive cells/organoid for the 1 Gy dose, with a 95% CI = 0.61, 2.21; *p*-value < 0.0001, and by 1.78 foci-positive cells/organoid for the 0.1 Gy dose, with a 95% CI = 1.03, 2.54; *p*-value < 0.0001). 

### 3.4. Involucrin Staining Reveals Dose-Dependent Decrease

**Figure 5:**
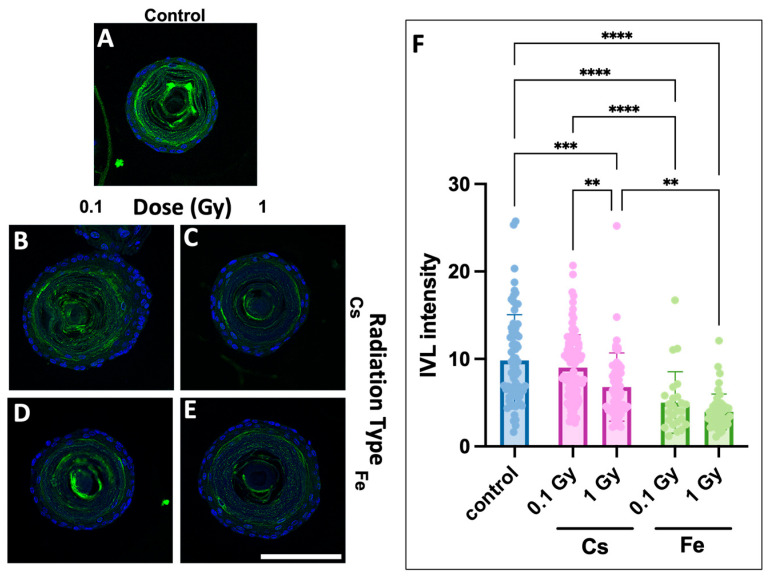
Involucrin staining of mouse organoids reveals dose- and radiation quality-dependent changes. Mouse organoids harvested on day 11 following initiation and day 9 post-radiation were sectioned to slides and stained and scored for levels of involucrin ((**A**–**E**). IVL is stained green with FITC, and nuclear DNA is counterstained with DAPI). A representative image of control (**A**), 0.1 Gy Cs (**B**), 1 Gy Cs (**C**), 0.1 Gy Fe (**D**) and 1 Gy Fe (**E**) are shown. Relative levels of fluorescence were then quantified and graphed (**F**). Error bars indicate std. devs. ** *p*-value < 0.01, *** *p*-value < 0.001, **** *p*-value < 0.0001. Scale bar, 100 µm.

We next performed immunofluorescence for Involucrin (IVL), a marker of squamous cell differentiation. Control and radiation-treated organoid sections were stained using anti-IVL antibody, and relative signal levels were quantified. From these studies, we observed a dose-dependent decrease in IVL staining, with the most severe effects being seen following Fe ion exposure (Figure 5). Significant differences were noted between the control and each dose, as well as between Cs and Fe ion-exposed organoids, with an increase in dose or LET resulting in a lower level of IVL expression. These findings suggest that LET suppresses terminal differentiation of esophageal keratinocytes, in agreement with the expansion of basaloid cells and intermediate differentiating (spinous) cell layers, but not cornifying superficial cell layers (Figure 2).

## 4. Discussion

**Figure 6:**
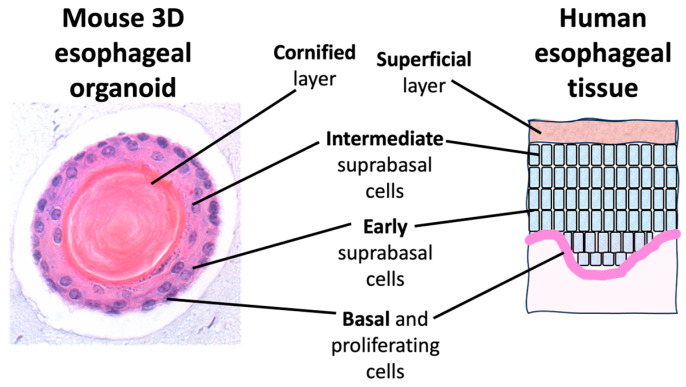
Relationship between layers of mouse esophageal organoid tissue section and human esophageal tissue section. Preliminary data suggest that radiation induced changes in the intermediate differentiating (spinous) layer, causing it to be thicker in the treated organoids.

These studies reveal that esophageal 3D organoids can be useful experimental systems for modeling the impact of radiation on esophageal tissue in vivo. We used both a low (0.1 Gy) and high (1 Gy) dose of two different radiation qualities to define persistent effects in our 3D organoid model system. In this study, cells were seeded on day one and irradiation exposure occurred on day two, prior to completion of organoid structures. Thus, the effects observed were due to persistent effects on cells that continued to divide and form organoids following each dose and radiation quality exposure. Our studies indicate that high-LET Fe 600 MeV/µm radiation is more damaging as compared to low-LET Cs radiation at an equal dose, as would be expected due to the greater deposition of energy from high-LET exposures. We surmise a greater damage induction by Fe ion exposure based upon the observed greater thickness of the organoid outer cell layers (composed of the intermediate suprabasal, early suprabasal, and basal proliferating cells; Figure 6), indicating an increase in proliferation in one of these layers. In addition, Fe-exposed organoids revealed higher levels of some key stress phospho-protein signaling molecules (JNK, ERK/MAPK, and p38) and persistent DNA damage levels. However, surprisingly, as shown in Figure 2D, a very similar effect in terms of a significant change in the thickness of the organoid cell layers was observed for both the lower 0.1 Gy dose and the 1 Gy dose of Fe ions. Based on the fluence and the size of the cells, we would expect ~36% of the cells to be hit at the lower 0.1 Gy dose and 99% of the cells to be hit at the 1 Gy dose. But even given the fact that many cells were not directly hit at the lower dose, a very similar impact on organoid morphology was observed as compared to the high dose. This finding may indicate that some sort of a bystander effect was at work in organoids exposed to the lower dose, in which neighboring unhit cells were impacted by exposure [24]. To provide a preliminary understanding of the defect induced by radiation, random images of Fe ion-exposed organoids were analyzed by a pathologist, who determined that the intermediate differentiating (spinous) layer was thicker in the treated organoids. Given that IVL is a marker for keratinocyte differentiation, we also stained control and treated mouse esophageal organoid sections with IVL to shed light upon the findings. We observed a dose- and radiation quality-dependent decrease in IVL staining (Figure 5), indicating that radiation can affect the differentiation process and that dose and LET status impact this process. Previous studies have revealed that downregulation of filaggrin and IVL is associated with barrier dysfunction [25]. Furthermore, previous investigations demonstrated that NOTCH signaling was augmented in lung basal stem cells upon radiation exposure. The authors postulated that ROS and DNA damage might trigger the NOTCH signaling feedback loop, leading to cellular differentiation to protect the long-lived stem cells from accumulating DNA mutations [26]. In the current study, Cs and Fe exposure appeared to perturb squamous cell differentiation by increasing the suprabasal (spinous) cell populations within the organoids (Figure 2). Given the critical role of NOTCH signaling in esophageal squamous epithelial differentiation and homeostasis [27], it would be warranted to explore the role of NOTCH signaling in esophageal 3D organoids treated with both low- and high-LET radiation.

Of particular interest was the increased stress phospho-protein expression for several phospho-proteins (pATF2, HSP27, MEK1, Msk1, and c-Jun) at the lower 0.1 Gy dose as compared to the higher 1 Gy dose for both Cs and Fe ion exposure (Figure 3). It is possible that a greater level of cell death may have occurred following the higher doses soon after exposure and that there was less cell killing at the lower dose, which then resulted in a higher level of expression at lower doses, given that the expression levels were obtained 9 days post-exposure. However, this was not the case for all phospho-proteins investigated, with some, such as Jnk, Erk, and p38, showing a greater induction at the higher dose following Fe ion exposure. Further time-course experiments would be needed to better define if some cell loss occurred at early timepoints. Moreover, phospho-Erk/MAPK was increased in cells exposed to Fe, but not in cells exposed to Cs, suggesting radiation quantity- as well as dose-dependent differential effects upon certain stress-induced signaling molecules. Additionally, an increase in p53 was detected but not statistically significant for Fe, warranting further investigation of the possibility that distinct radiation qualities differentially influence the post-radiation kinetics of stress-induced signaling molecules as well as p53 protein stability. However, given the persistent stress phospho-protein expression at the lower doses, it is of concern that even lower doses may lead to persistent changes, which, over time impact tissue health. 

Given that these effects were measured and observed nine days post-exposure, when the initial DNA DSBs induced should have been repaired, the persistent changes in the exposed cells induced by the radiation exposure may indicate hyperactive DNA replication or other metabolic changes that accumulate endogenous DNA-damaging agents such as aldehydes. Reactive oxygen species (ROS) are often implicated in persistent changes following radiation exposure and known to persist for long periods and to lead to genomic instability. Although, in this study, we were not able to quantify levels of ROS, a previous study by Werner et al. [27] revealed that high-LET radiation is a more effective inducer of p38 and that the induction of p38 can block ROS production and lead to genomic instability. The high levels of p38 that block ROS may have unintended consequences, as moderate levels of ROS are essential for normal cellular function and signaling, and thus the lack of such signaling may disrupt the cell’s ability to counteract these stressors. The work by Werner et al. further supported this model when they altered the redox status of cells towards increased ROS levels by interference with p38MAPK or inhibiting catalase activity and observed reduced genomic instability. Our findings of much greater p38 activation in high-LET exposed organoids and higher levels of persistent DNA damage are consistent with these previous observations. Based on our findings and this previously published work, we speculate that high-LET Fe ion exposure induces p38 activation, which in turn inhibits ROS induction and leads to an increase in genomic instability through a loss of homeostasis. Future studies would benefit from the inclusion of the quantification of ROS levels in exposed organoids to further verify our hypothesis. 

The observed alterations in esophageal tissue morphology and sustained activation of stress phospho-protein signaling at relatively low doses present intriguing findings. It is imperative, however, to acknowledge the experimental context wherein a mouse 3D esophageal organoid system was employed. While such models offer valuable insights into the effects of diverse exposures, validating these outcomes in human systems necessitates additional investigations. To ascertain the translational relevance of our observations, further in vivo studies employing mouse models and human-derived cells are warranted. While esophageal 3D organoid models provide a robust platform for preliminary investigations, extrapolating these findings to human physiology demands comprehensive validation through in vivo experimentation.

In summary, even fairly low doses of high-LET radiation (0.1 Gy) can induce epithelial changes in combination with genomic instability and may increase the risk of cancer. Additionally, the observed high activation of p38 MAPK signaling warrants further study to evaluate the utility of phospho-p38 as a biomarker for radiation-induced cancer risk. Lastly, persistently elevated higher levels of various phospho-proteins within the stress pathway were observed following low doses (0.1 Gy) of both high- and low-LET radiation, and this chronic signaling may result in changes in the cellular microenvironment and potentiate cancer.

## Data Availability

Data are contained within the article.
